# The relationship between loneliness and blood glucose: a cross-sectional survey among Japanese

**DOI:** 10.1186/s13104-024-06855-z

**Published:** 2024-07-22

**Authors:** Quyen An Tran, Sho Nakamura, Kaname Watanabe, Choy-Lye Chei, Hiroto Narimatsu

**Affiliations:** 1 Graduate School of Health of Innovation, Kanagawa University of Human Services, Kawasaki, Kanagawa Japan; 2 Cancer Prevention and Control Division, Kanagawa Cancer Center Research Institute, 2-3-2 Nakao, Asahi-ku, Yokohama, Kanagawa 241-8515 Japan; 3 Department of Genetic Medicine, Kanagawa Cancer Center, Yokohama, Japan

**Keywords:** Loneliness, Diabetes, HbA1c, Socioeconomic status

## Abstract

Recently, researchers have uncovered a correlation between loneliness and both the development and management of diabetes. Nevertheless, previous studies employing an unvalidated loneliness questionnaire impair result accuracy. Furthermore, this aspect has not been researched in the Japanese population. Therefore, this cross-sectional study analyzed data from the Kanagawa prospective “ME-BYO” Cohort Study (ME-BYO cohort) to investigate the correlation between loneliness, as measured by 20 items on the UCLA Loneliness Scale, and blood glucose levels. A total of 666 participants were included in the analysis, with a mean age of 54.1 years and a mean BMI of 23 kg/m^2^. Half of the participants had obtained an education level beyond high school. The mean household income and physical activity level were reported as 6.83 million Japanese yen and 12.3 METs-h/day, respectively. Model 1 of the linear regression analysis determined that there was no significant association between the loneliness scale and HbA1c (p = 0.512). After adjusting for age, gender, BMI (model 2), sitting time, physical activity level (model 3), housemates, household income, and final education (model 4), and controlling for social support, quality of life, and depression (model 5), the results showed no significant association, with a p-value of 0.823, 0.791, 0.792, and 0.816, respectively. Thus, the study found no link between loneliness and HbA1c in the high SES population. This finding contradicts previous results and may be attributed to the impact of population characteristics, SES status, or genetic backgrounds.

**Supplementary Information:**

The online version contains supplementary material available at 10.1186/s13104-024-06855-z.

## Introduction

According to the International Diabetes Federation, in 2021, about 537 million people have diabetes worldwide; this number is projected to reach 643 million by 2030 and 783 million by 2045, while 45% of people with undiagnosed diabetes. It confirms that diabetes is one of the fastest-growing global health emergencies of the twenty-first century [1]. The growth of diabetes also has been observed in Japan. In 2009, it was reported that 13.5% of the Japanese population has either type 2 diabetes mellitus (T2DM) or impaired glucose tolerance, and 6% of the total Japanese healthcare budget was accounted for diabetes [2]. To prevent and manage T2DM, many issues have been considered, such as nutrition and physical activity [3]. Recently, psychosocial aspects have been emerging as one crucial matter directly affecting the onset and management of diabetes [4].

Regarding the psychosocial aspect, loneliness is becoming a common issue, as many as 80% of children and 40% of adults over 65 years old reported being lonely [5, 6]. It was reported that lonely people had 26% greater odds of early mortality than others. Loneliness is associated with an increased risk of cardiovascular disease, metabolic syndrome, functional disability, dementia, and mild cognitive impairment [7, 8]. Loneliness is defined as a negative feeling that occurs when an individual perceives that their social needs are not being met [9]. Although many instruments have been developed to assess loneliness in various contexts [10], such as the de Jong Gierveld Loneliness Scale [11] and the Campaign to End Loneliness Measurement Tool, loneliness is most widely measured using the University of California, Los Angeles Loneliness Scale (UCLA) [12]. This scale offers many advantages, including comprehensive measurement, high reliability and validity, and wide application [12, 13].

The relationship between loneliness and blood glucose has been gradually clarified. Loneliness has been observed to be associated with the elevation of cortisol levels [14], as well as the increase of inflammatory cytokines such as interleukin-6, interleukin-1, and monocyte chemotactic protein-1 [15]. Cortisol plays an important mechanical function related to glucose homeostasis, such as promoting gluconeogenesis in the liver and regulating glycogen metabolism. Thus, the increase in cortisol levels is associated with the elevation of plasma blood glucose [16]. A study has shown that raised diurnal cortisol is predictive of future glucose disturbances [17]. Additionally, the elevation of interleukin-6, interleukin-1, and monocyte chemotactic protein-1 in the body induced by loneliness, is a known risk factor for insulin resistance and the development of type 2 diabetes [18–20]. Moreover, loneliness has been demonstrated to significantly predict T2DM [21, 22] and be associated with bad diabetes management [23, 24].

Research on hospitalized diabetes patients showed that loneliness was associated with higher blood pressure but not correlated to other indicators of blood glucose control. This research was conducted in a group of patients admitted to the hospital with an indication for hospitalization. Thus, increased glucose levels were the reason for admission in half of the participants; it may be assumed that blood glucose control was unsatisfactory already at hospital admission in this group of patients [24]. Other research was longitudinally conducted in diabetes-free participants, showing that the onset of diabetes was significantly higher in the group with a higher loneliness score [21, 22]. Another study with a relatively small sample size of 92 diabetes out-patients showed that loneliness, assessed by Midlife in the United States—Refresher (MIDUS-R)—an incomplete version of the University of California, Los Angeles Loneliness Scale, was associated with HbA1c > 7% [23]. However, the MIDUS-R scale has not been validated. Therefore, the effect of loneliness on T2DM management, which HbA1c best represents, has not been clarified. Otherwise, in the Asian population, particularly Japanese, the direct relationship between loneliness and HbA1c has not been studied. It was reported that loneliness was higher in United States (22%) and United Kingdom (23%) than in Japan (9%) [25]. Additionally, Japanese people have different lifestyles, diets, and socioeconomic backgrounds compared to Western countries [26, 27]. Besides, glucose tolerance is acknowledged as significantly varying across regions and populations [1]. As a result, the relationship between loneliness and blood glucose may differ across populations. Therefore, we would like to conduct this research to elucidate the relationship between loneliness and blood glucose represented by HbA1c in the Japanese population. The result of this study can accumulate knowledge of the association between loneliness and blood glucose and blood glucose management, particularly in Japan. It would also provide important clues for basic studies to clarify this complex basic mechanism, which might involve cortisol and inflammatory cytokines.

## Material and methods

### Study design

The cross-sectional study was conducted as part of the Kanagawa Prospective “ME-BYO” Cohort Study (ME-BYO cohort), which formed part of a larger collaborative genomic cohort study, namely the Japan Multi-Institutional Collaborative Cohort Study (J-MICC Study) [28]. The baseline recruitment for the ME-BYO cohort began in 2016 and is still ongoing in 2023, encompassing participants aged 18–95 residing or working in Kanagawa Prefecture.

The structure of the baseline survey, described elsewhere [28], included a self-administered questionnaire and health examinations of the ME-BYO cohort. The questionnaire gathered information on various aspects of sociodemographic characteristics, health history, lifestyle, nutrition intake, and psychology. Additionally, physical check-ups and laboratory tests were also conducted, including blood chemistry and complete blood cell count data.

**Fig. 1:**
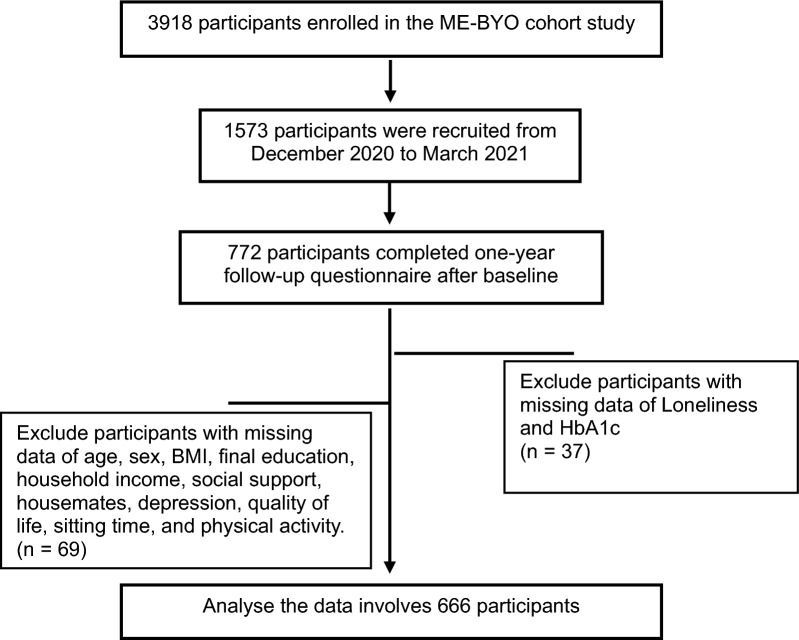
Participant’s selection process

The ME-BYO cohort study recruited 3918 participants as of March 2022. The loneliness data were obtained from 1573 participants recruited from December 2020 to March 2021. This study sampled 666 participants with sufficient data for loneliness, HbA1c, depression, social support, age, sex, BMI, final education, household income, housemates, quality of life, sitting time, and physical activity; the participant’s selection is presented in Fig. 1.

Demographic data included age, sex, final education level, household income, housemates, quality of life, sitting time, and physical activity. Final education levels were divided into three groups: low as high school or under high school, middle as vocational school or junior college, and high as university or higher. Housemates refer to the number of people living with the participant. We assessed loneliness with the 20-item of the University of California, Los Angeles (UCLA) Loneliness Questionnaire [29] which has also been translated into Japanese and validated in Japan [30] (response options 1 = never to 4 = always); total scores ranged from 20 to 80; higher scores indicated more severity and higher frequency of loneliness. Psychological distress was evaluated by the 6-item Kessler Psychological Distress Scale (K6) score, a robust non-specific psychological distress measurement tool [31, 32]. K6 score is calculated from 6 items using a 5-point Likert scale numbered 1–5, with a total score ranging from 6 to 30; a higher score indicates more severe distress. We used a Japanese version of scale [31], translated and validated from the original scale developed in English [32]. Social support was evaluated by the ENRICHD Social Support Instrument, a seven-item using a 5-point Likert scale numbered 1–5, the self-report measure used in the ENRICHD trial [33]. Individual items are then summed for a total score, with higher scores indicating greater social support. Based on a previously reported method, the daily physical activity was calculated as metabolic equivalents-hours per day (METs-h/day) [34]. In other words, daily life activity and leisure-time activity were measured based on the International Physical Activity Questionnaire [35]. The intensity of activity was divided into five categories: walking (3.0 METs), heavy physical work or exercise (4.5 METs) during daily life activities, and leisure-time activities with intensity levels of 3.4, 7.0, and 10.0 METs. To calculate intensity (METs-h/day), we multiplied the intensity by the duration and frequency of each activity. Standing time (2.0 METs) was not included in our analysis as we focused on assessing moderate-to-vigorous physical activity (> 3METs). The amount of time spent sitting (h/day) was obtained from the questionnaire [36]. The outcome is HbA1c obtained from blood samples collected in the baseline survey, which is reported as a percentage of total hemoglobin; higher HbA1c indicates worse blood glucose management [37].

All research procedures were approved by the Institutional Review Board of Kanagawa Cancer Center (28KEN-36). Written informed consent was obtained from all participants for the ME-BYO cohort.

### Statistical analysis

All statistical analyses were performed with R (Version 4.1.0; R Core Team, Vienna, Austria) [38]. Descriptive, Chi-square, and t-tests were used to describe and compare differences between two groups of non-diabetes participants with HbA1c < 6.5%, and the group of diabetes participants with HbA1c ≥ 6.5% or diagnosed as diabetes or using oral diabetes medicine. Correlation analysis was conducted to examine the association between variables. Multiple linear regression is used to assess the association between loneliness and HbA1c in 617 participants who have not been diagnosed with diabetes. Step 1 assessed the association between loneliness and HbA1c as model 1. Step 2 entered age, gender, and BMI as model 2. Step 3 entered sitting time, and physical activity as model 3. Step 4 entered housemates, household income, and final education as model 4. Step 5 entered social support, quality of life, and depression as model 5. The association between loneliness and HbA1c is also assessed in subgroup analysis for low and high income at cut-off point 400 × 10^4^JPY [39], for final education as low educational level versus middle and high educational level, and for physical activity level at the cut-off point 6 METs-h/day.

## Results

The mean (standard deviation [SD]) age and BMI of the 666 participants in this study are 54.1 (13.4) years and 23 (3.4) kg/m^2^, respectively. Of those, 335/666 (50%) had an educational level higher than high school. Almost 90% (586/666) of participants lived with at least one housemate. Participants reported moderate social support at a mean (SD) of 26.5 (5.5), low depressive symptoms at a mean (SD) of 4.2 (4.3), and moderate loneliness at a mean (SD) of 51 (4.9). The mean HbA1c was 5.6 (SD 0.6, range 4.6–11.0); 7.8% (52/666) of participants were persons with diabetes (Table 1).

**Table 1:** Participant characteristics

Characteristic	Overall (N = 666^1^)	Non-Diabetes (N = 614^1^)	Diabetes (N = 52^1^)	p-value^2^
Age	54.1 (13.4)	53.3 (13.3)	64.1 (10.2)	< 0.001
BMI	23.0 (3.4)	22.9 (3.3)	25.0 (3.8)	< 0.001
Gender				0.062
Female	326/666 (49%)	307/614 (50%)	19/52 (37%)	
Male	340/666 (51%)	307/614 (50%)	33/52 (63%)	
Final education				0.60
High education	335/666 (50%)	308/614 (50%)	27/52 (52%)	
Mid education	151/666 (23%)	142/614 (23%)	9/52 (17%)	
Low education	180/666 (27%)	164/614 (27%)	16/52 (31%)	
Household income (10.000 JPY)	683.8 (407.0)	693.5 (411.9)	569.5 (326.4)	0.026
Housemates				0.003
0	80/666 (12%)	72/614 (12%)	8/52 (15%)	
1	266/666 (40%)	236/614 (38%)	30/52 (58%)	
2	299/666 (45%)	288/614 (47%)	11/52 (21%)	
3	20/666 (3.0%)	17/614 (2.8%)	3/52 (5.8%)	
4	1/666 (0.2%)	1/614 (0.2%)	0/52 (0%)	
Social support	26.5 (5.5)	26.6 (5.5)	25.6 (6.3)	0.33
Depression	4.2 (4.3)	4.2 (4.3)	4.6 (4.1)	0.43
Loneliness	51.0 (4.9)	51.0 (5.0)	51.8 (4.3)	0.29
Quality of life	0.9 (0.1)	0.9 (0.1)	0.9 (0.1)	0.004
Physical activity (METs-h/day)	12.3 (11.2)	12.4 (11.4)	11.4 (9.2)	0.75
Sitting time (hours)	4.2 (2.8)	4.2 (2.8)	4.1 (2.6)	0.89
HbA1c (%)	5.6 (0.6)	5.5 (0.4)	7.1 (0.9)	< 0.001

^1^Mean (SD); n/N (%)

^2^Wilcoxon rank sum test; Pearson’s Chi-squared test; Fisher’s exact test

HbA1c is positively correlated with age r(615) = 0.27 (95% confidence interval [CI] (0.19, 0.34), p < 0.01) and BMI r(615) = 0.26 (95% CI (0.18, 0.33), p < 0.01). Depression is strongly correlated with loneliness r(615) = 0.24 (95% CI (− 0.19, − 0.04), p < 0.01) (Table 2).

**Table 2:** Correlation between study variables

Variable	Mean	SD	1	2	3	4	5	6	7	8	9	10
1. HbA1c	5.54	0.43										
2. Age	53.35	13.37	0.27**									
			[0.19, 0.34]									
3. BMI	22.93	3.34	0.26**	0.06								
			[0.18, 0.33]	[− 0.02, 0.14]								
4. Household income	687.90	412.83	− 0.08	− 0.15**	0.03							
			[− 0.16, 0.00]	[− 0.23, − 0.07]	[− 0.05, 0.11]							
5. Housemates	1.41	0.74	0.01	− 0.06	0.05	0.31**						
			[− 0.07, 0.09]	[− 0.14, 0.02]	[− 0.03, 0.13]	[0.24, 0.38]						
6. Social support (ESSI)	26.65	5.42	0.02	0.05	− 0.05	0.15**	0.27**					
			[− 0.06, 0.10]	[− 0.03, 0.13]	[− 0.13, 0.02]	[0.07, 0.23]	[0.19, 0.34]					
7. Sitting time	4.19	2.87	− 0.02	− 0.07	0.02	0.06	− 0.04	0.02				
			[− 0.10, 0.06]	[− 0.15, 0.00]	[− 0.06, 0.09]	[− 0.02, 0.14]	[− 0.12, 0.04]	[− 0.06, 0.10]				
8. Physical activity	12.41	11.33	− 0.00	− 0.02	− 0.05	− 0.02	− 0.04	− 0.00	− 0.28**			
			[− 0.08, 0.08]	[− 0.10, 0.06]	[− 0.13, 0.03]	[− 0.10, 0.05]	[− 0.12, 0.04]	[− 0.08, 0.07]	[− 0.35, − 0.20]			
9. Depression (K6)	4.19	4.32	− 0.04	− 0.19**	0.00	− 0.05	− 0.01	− 0.32**	0.03	0.09*		
			[− 0.12, 0.04]	[− 0.26, − 0.11]	[− 0.08, 0.08]	[− 0.12, 0.03]	[− 0.09, 0.07]	[− 0.39, − 0.25]	[− 0.05, 0.11]	[0.01, 0.17]		
10. Quality of life (EQ-5D-5L)	0.93	0.10	− 0.07	− 0.09*	− 0.06	0.15**	0.02	0.20**	0.01	− 0.05	− 0.41**	
			[− 0.15, 0.00]	[− 0.16, − 0.01]	[− 0.14, 0.02]	[0.07, 0.23]	[− 0.06, 0.10]	[0.13, 0.28]	[− 0.07, 0.09]	[− 0.13, 0.03]	[− 0.47, − 0.34]	
11. Loneliness (UCLA)	50.98	4.98	− 0.03	− 0.04	− 0.05	− 0.06	− 0.04	− 0.11**	0.02	0.08	0.24**	− 0.11**
			[− 0.11, 0.05]	[− 0.12, 0.03]	[− 0.13, 0.03]	[− 0.13, 0.02]	[− 0.12, 0.04]	[− 0.19, − 0.03]	[− 0.06, 0.10]	[− 0.00, 0.15]	[0.17, 0.32]	[− 0.19, − 0.04]

617 participants were involved in this analysis. Value are presented as mean ± standard deviation. Values in square brackets indicate the 95% confidence interval. * indicates p < 0.05. ** indicates p < 0.01

In the linear regression analysis (Table 3), the loneliness scale was not significantly associated with HbA1c (p = 0.512). The association between the loneliness scale and HbA1c was not evident in previously described regression models 2–5. The lowest p-value among the four models was 0.791.

**Table 3:** Linear regression model of HbA1c

	HbA1c (Model 1)		HbA1c (Model 2)		HbA1c (Model 3)		HbA1c (Model 4)		HbA1c (Model 5)	
Predictors	Estimates (95% CI)	p	Estimates (95% CI)	p	Estimates (95% CI)	p	Estimates (95% CI)	p	Estimates (95% CI)	p
(Intercept)	5.652	** < 0.001**	4.274	** < 0.001**	4.265	** < 0.001**	4.416	** < 0.001**	4.506	** < 0.001**
	5.299–6.005		3.840–4.708		3.826–4.704		3.943–4.889		3.881–5.130	
Loneliness	− 0.002	0.512	− 0.001	0.823	− 0.001	0.791	− 0.001	0.792	− 0.001	0.816
	− 0.009–0.005		− 0.007–0.006		− 0.007–0.006		− 0.007–0.006		− 0.007–0.006	
Age			0.008	** < 0.001**	0.008	** < 0.001**	0.008	** < 0.001**	0.008	** < 0.001**
			0.006–0.011		0.006–0.011		0.006–0.011		0.005–0.011	
Female			0.092	**0.007**	0.093	**0.007**	0.071	**0.048**	0.066	0.071
			0.025–0.159		0.026–0.160		0.001–0.142		− 0.006–0.139	
BMI			0.035	** < 0.001**	0.035	** < 0.001**	0.034	** < 0.001**	0.033	** < 0.001**
			0.025–0.045		0.025–0.045		0.023–0.044		0.023–0.043	
Sitting time					0.000	0.990	0.002	0.800	0.001	0.822
					− 0.012–0.012		− 0.010–0.013		− 0.010–0.013	
Physical activity					0.001	0.592	0.001	0.700	0.001	0.734
					− 0.002–0.004		− 0.002–0.004		− 0.002–0.004	
Housemates							0.015	0.522	0.012	0.637
							− 0.031–0.061		− 0.037–0.060	
Household income							− 0.000	0.692	− 0.000	0.717
							− 0.000–0.000		− 0.000–0.000	
Final education							− 0.025	**0.045**	− 0.025	**0.045**
							− 0.050 to − 0.001		− 0.050 to − 0.001	
Social Support									0.002	0.652
									− 0.050–0.008	
Quality of life									− 0.117	0.517
									− 0.472–0.238	
Depression									− 0.001	0.865
									− 0.010–0.008	
Observations	617		617		617		617		617	
R^2^ /R^2^ adjusted	0.001/− 0.001		0.147/0.140		0.147/0.137		0.147/0.135		0.148/0.131	

Bold numbers indicate statistical significance

*CI* Confidence interval, *BMI* Body mass index

The subgroup analysis was performed and the result is shown in the supplemental tables; the association between loneliness and HbA1c still has not been found with all p-values > 0.05. In the group with income < 400 × 10^4^JPY (Table S1), after adjusting by co-variables in (model 2), (model 3), and (model 4), the association failed to be detected with all p-values > 0.05. In the group with income > 400 × 10^4^JPY, similarly to the cohort of income ≤ 400 × 10^4^JPY, after adjusting for co-variables, the association again failed to be detected with all p-values > 0.05. Table S2 shows the result of subgroup analysis when splitting the population into low and middle-education group and high-education group. After adjusting for co-variables in (model 2), (model 3), and (model 4), the association between loneliness and HbA1c has failed to detect, with all p-values > 0.05. Table S3 shows the linear regression analysis result on the group has physical activity level > 6 METs-h/day and ≤ 6 METs-h/day, respectively. After adjusting for co-variables in (model 2), (model 3), (model 4), the association between loneliness and HbA1c still has not been detected, all p-values > 0.05.

## Discussion

The association between loneliness and HbA1c has not been found in this study. Although this is a cross-sectional study in which a definite causal relationship has to be investigated in further study, it suggests that loneliness may not be associated with the risk of T2DM. Notably, loneliness was closely associated with social capital and socioeconomic status (SES) [40–42]. Besides, SES was reported to directly or indirectly affect the risk of T2DM [43]. However, this study indicates that in high SES people, loneliness may not be associated with the risk of T2DM. This means the intervention or management against being lonely would not prevent the onset of T2DM.

Our finding is not consistent with previous reports on Western populations, which suggests that the impact of loneliness in the Asian population, specifically Japanese in this study, may be different from that in Western countries [21–24, 35]. Some reasons can explain this inconsistency. Firstly, the participants’ HbA1c levels were less diverse than in previous studies. This study’s participants have a mean HbA1c of 5.6% with a narrow standard deviation of 0.6, and over 95% of participants have HbA1c < 6.5 (Table 1). Also, the percentage of loneliness is lower in the Japanese population than in Western countries [25]. Thus, statistical power is possibly too small to detect the effect of loneliness. Future longitudinal analysis of the ME-BYO cohort is warranted for further assessment. Secondly, the participants’ SES is higher in this study than that of the normal Japanese population, which can be explained as follows. The household income in this study (683.8 ± 407.0 × 10^4^JPY) is over 1.5 times higher than the average income of the Japanese population [39, 44]. Additionally, the percentage of participants who attain an educational level higher than high school is 50% (Table 2), which is over three times higher than this rate of the older Japanese population [39, 45]. Based on these characteristics, participants of our study can be considered high SES in favorable health status. It was proven that the high SES population has less risk of diabetes compared to the low SES population [40, 41]. Therefore, in the high SES population, loneliness may have less impact on diabetes. People with high SES usually have healthy diets and enough knowledge to maintain good health. In this study, the physical activity was observed at a mean of 12.3 METs-h/day, equal to 86.1 METs-h/week, almost 3.7 times higher than the Japanese reference value of 23 METs-h/week [46]. In addition, this study population has a mean BMI of 23 ± 3.4 kg/m^2^, lower than that of other studies (27.5 ± 4.6 kg/m^2^) in the UK and (26.8 ± 4.9) in Ireland [21, 47]. Thus, loneliness possibly does not affect the HbA1c of Japanese people in this study. Although the status of loneliness does not significantly impact HbA1c even in the lower SES group (Table S1, S2, S3), we still need to investigate this impact on HbA1c studies, including the participants with lower SES compared to the Japanese general population. Lastly, unrecognized or uncollected factors might have affected this inconsistency. Support from the community and family is important to prevent T2DM. The way of living or characteristics of communities is different between Japan and Western countries, which may affect the relationship between loneliness and T2DM [48, 49]. However, such studies investigating these differences from this viewpoint are extremely limited. The difference in genetic backgrounds also may affect the result. This may be veiled in gene-environmental interactions, which is still unknown [50]. Thus, future studies investigating these points mentioned above would give us a proper interpretation.

### Limitations

This study has provided a novel finding about the relationship between T2DM and loneliness in the population with high SES and healthy life. We utilized the fully validated questionnaire, the entire UCLA Loneliness scale, which solved the limitations of the previous US study that used an unvalidated questionnaire [23]. However, some limitations remain to be discussed. The finding of this study can be applied solely to the Japanese population, as the association between loneliness and blood glucose may differ across populations. The sample size (n = 666) was relatively small, and there was a low rate of diabetes participants, resulting in an undiversified population caused by selection bias. To address these limitations, future studies should focus on diversifying and broadening the study population to include individuals with low SES and varying health conditions. According to the sample size calculation, the required sample size varies based on the correlation coefficient between loneliness and blood glucose. Although sample size of our study is inadequate, it provide a new finding as abovementioned. Additionally, relying solely on participants’ self-reports to assess loneliness status may introduce recall bias or socially desirable responses, which could influence the accuracy of the findings. Alternative solutions, such as face-to-face interviews, should be considered to mitigate these issues. This study solely focuses on investigating the direct relationship between loneliness and HbA1c, without assessing the complex underlying mechanisms involving cortisol and various inflammatory cytokines. Addressing these mechanism would have required a more extensive and invasive data-collection process beyond the scope of our study. Although this study did not include data on such biomarkers, the findings provide important clues for future research on the basic mechanisms. Lastly, this cross-sectional study cannot establish a cause-effect relationship between loneliness and diabetes. A longitudinal study design should be implemented in future research to explore causality.

## Conclusion

In conclusion, the association between loneliness and HbA1c has not been observed in the high SES population of this study. Although our study did not find any evidence of an association between HbA1c and loneliness, it is essential to investigate the association in the lower SES Japanese populations. Extending the longitudinal study of the ME-BYO cohort is needed to conclude the mechanism between blood glucose management and loneliness.

### Supplementary Information

Supplementary Material 1.

## Data Availability

The datasets used and/or analyzed during the current study are available from the corresponding author upon reasonable request. Data from the “Japan Multi-Institutional Collaborative Cohort Study and the ME-BYO cohort” are not publicly available due to participants’ privacy concerns.

## Abbreviations

UCLA: University of California, Los Angeles
BMI: Body mass index
METs-h/day: Metabolic equivalent tasks hour per day
HbA1c: Hemoglobin A1c
SES: Socioeconomic status
MIDUS-R: Midlife in the United States Refresher
T2DM: Type 2 diabetes mellitus
