# Supplementary material for: The relationship between loneliness and blood glucose: a cross-sectional survey among Japanese
Source: BMC Res Notes. 2024 Jul 22;17:201. doi: 10.1186/s13104-024-06855-z (PMC11264398; doi:10.1186/s13104-024-06855-z)
Supplement: Supplementary file 1 — Supplementary Material 1. [file 13104_2024_6855_MOESM1_ESM.docx]

|  | ***HbA1c (Model 1)*** | | | ***HbA1c (Model 2)*** | | | ***HbA1c (Model 3)*** | | | ***HbA1c (Model 4)*** | | | |
| --- | --- | --- | --- | --- | --- | --- | --- | --- | --- | --- | --- | --- | --- |
| ***Predictors*** | ***Est*** | ***(95% CI)*** | ***p*** | ***Est*** | ***(95% CI)*** | ***p*** | ***Est*** | ***(95% CI)*** | ***p*** | ***Est*** | ***(95% CI)*** | ***p*** |  |
| ***Income < 400 x 103 (JPY)*** | | | | | | | | | | | | | |
| **(Intercept)** | 5.698 | 5.031 – 6.365 | **<0.001** | 4.312 | 3.538 – 5.085 | **<0.001** | 4.301 | 3.513 – 5.089 | **<0.001** | 4.711 | 3.552 – 5.869 | **<0.001** |  |
| **Loneliness** | -0.002 | -0.015 – 0.011 | 0.723 | 0.002 | -0.009 – 0.014 | 0.695 | 0.002 | -0.010 – 0.013 | 0.754 | 0.000 | -0.012 – 0.013 | 0.944 |  |
| **Age** |  |  |  | 0.012 | 0.008 – 0.016 | **<0.001** | 0.012 | 0.008 – 0.016 | **<0.001** | 0.012 | 0.008 – 0.016 | **<0.001** |  |
| **Female** |  |  |  | 0.042 | -0.087 – 0.172 | 0.520 | 0.048 | -0.084 – 0.179 | 0.473 | 0.058 | -0.080 – 0.196 | 0.409 |  |
| **BMIcom** |  |  |  | 0.020 | 0.004 – 0.036 | **0.012** | 0.020 | 0.004 – 0.036 | **0.012** | 0.018 | 0.001 – 0.034 | **0.036** |  |
| **Sitting time** |  |  |  |  |  |  | 0.008 | -0.014 – 0.029 | 0.493 | 0.007 | -0.016 – 0.029 | 0.544 |  |
| **Physical activity** |  |  |  |  |  |  | -0.000 | -0.005 – 0.005 | 0.955 | -0.000 | -0.006 – 0.005 | 0.966 |  |
| **Housemates** |  |  |  |  |  |  |  |  |  | 0.032 | -0.055 – 0.120 | 0.463 |  |
| **Final education** |  |  |  |  |  |  |  |  |  | 0.007 | -0.035 – 0.049 | 0.726 |  |
| **Social support** |  |  |  |  |  |  |  |  |  | -0.004 | -0.016 – 0.009 | 0.578 |  |
| **Quality of life** |  |  |  |  |  |  |  |  |  | -0.285 | -0.890 – 0.321 | 0.354 |  |
| **Depression** |  |  |  |  |  |  |  |  |  | -0.001 | -0.016 – 0.014 | 0.900 |  |
| **Observations** | 138 | | | 138 | | | 138 | | | 138 | | | |
| **R^2^ / R^2^ adjusted** | 0.001 / -0.006 | | | 0.260 / 0.238 | | | 0.263 / 0.230 | | | 0.276 / 0.213 | | | |
| ***Income >= 400 x 103 (JPY)*** | | | | | | | | | | | | | |
| **(Intercept)** | 5.656 | 5.242 – 6.071 | **<0.001** | 4.256 | 3.740 – 4.772 | **<0.001** | 4.258 | 3.736 – 4.780 | **<0.001** | 4.503 | 3.762 – 5.244 | **<0.001** |  |
| **Loneliness** | -0.003 | -0.011 – 0.005 | 0.524 | -0.002 | -0.010 – 0.006 | 0.589 | -0.002 | -0.010 – 0.005 | 0.542 | -0.003 | -0.011 – 0.005 | 0.496 |  |
| **Age** |  |  |  | 0.007 | 0.004 – 0.010 | **<0.001** | 0.007 | 0.004 – 0.010 | **<0.001** | 0.006 | 0.003 – 0.009 | **<0.001** |  |
| **Female** |  |  |  | 0.111 | 0.031 – 0.191 | **0.007** | 0.114 | 0.034 – 0.194 | **0.005** | 0.074 | -0.013 – 0.161 | 0.095 |  |
| **BMIcom** |  |  |  | 0.042 | 0.030 – 0.054 | **<0.001** | 0.042 | 0.030 – 0.055 | **<0.001** | 0.039 | 0.027 – 0.052 | **<0.001** |  |
| **Sitting time** |  |  |  |  |  |  | -0.003 | -0.016 – 0.011 | 0.71 | -0.002 | -0.015 – 0.012 | 0.812 |  |
| **Physical activity** |  |  |  |  |  |  | 0.001 | -0.002 – 0.005 | 0.416 | 0.001 | -0.003 – 0.004 | 0.61 |  |
| **Housemates** |  |  |  |  |  |  |  |  |  | 0.002 | -0.055 – 0.059 | 0.941 |  |
| **Final education** |  |  |  |  |  |  |  |  |  | -0.036 | -0.065 – -0.007 | **0.016** |  |
| **Social support** |  |  |  |  |  |  |  |  |  | 0.003 | -0.005 – 0.011 | 0.454 |  |
| **Quality of life** |  |  |  |  |  |  |  |  |  | -0.06 | -0.494 – 0.374 | 0.787 |  |
| **Depression** |  |  |  |  |  |  |  |  |  | 0.001 | -0.010 – 0.012 | 0.813 |  |
| **Observations** | 479 | | | 479 | | | 479 | | | 479 | | | |
| **R^2^ / R^2^ adjusted** | 0.001 / -0.001 | | | 0.123 / 0.116 | | | 0.125 / 0.114 | | | 0.137 / 0.117 | | | |

Table S1: Linear Regression Model of HbA1c (Subgroup for Household Income)

Table S2: Linear Regression Model of HbA1c (Subgroup for Education)

|  | ***HbA1c (Model 1)*** | | | ***HbA1c (Model 2)*** | | | ***HbA1c (Model 3)*** | | | ***HbA1c (Model 4)*** | | |
| --- | --- | --- | --- | --- | --- | --- | --- | --- | --- | --- | --- | --- |
| ***Predictors*** | ***Est*** | ***(95% CI)*** | ***p*** | ***Est*** | ***(95% CI)*** | ***p*** | ***Est*** | ***(95% CI)*** | ***p*** | ***Est*** | ***(95% CI)*** | ***p*** |
| ***Low and Mid Education*** | | | | | | | | | | | | |
| **(Intercept)** | 6.057 | 5.492 – 6.622 | **<0.001** | 4.496 | 3.778 – 5.214 | **<0.001** | 4.568 | 3.844 – 5.293 | **<0.001** | 4.694 | 3.715 – 5.673 | **<0.001** |
| **Loneliness** | -0.009 | -0.020 – 0.002 | 0.110 | -0.005 | -0.016 – 0.005 | 0.301 | -0.005 | -0.016 – 0.005 | 0.334 | -0.004 | -0.015 – 0.006 | 0.415 |
| **Age** |  |  |  | 0.008 | 0.004 – 0.012 | **<0.001** | 0.008 | 0.004 – 0.012 | **<0.001** | 0.008 | 0.003 – 0.012 | **0.001** |
| **Female** |  |  |  | 0.029 | -0.088 – 0.147 | 0.623 | 0.031 | -0.087 – 0.148 | 0.61 | 0.009 | -0.113 – 0.132 | 0.88 |
| **BMI** |  |  |  | 0.041 | 0.025 – 0.057 | **<0.001** | 0.040 | 0.024 – 0.056 | **<0.001** | 0.04 | 0.023 – 0.056 | **<0.001** |
| **Sitting time** |  |  |  |  |  |  | -0.016 | -0.037 – 0.004 | 0.124 | -0.017 | -0.039 – 0.004 | 0.105 |
| **Physical activity** |  |  |  |  |  |  | -0.001 | -0.005 – 0.004 | 0.758 | -0.001 | -0.005 – 0.004 | 0.744 |
| **Household income** |  |  |  |  |  |  |  |  |  | 0.000 | -0.000 – 0.000 | 0.414 |
| **Housemates** |  |  |  |  |  |  |  |  |  | -0.001 | -0.081 – 0.080 | 0.983 |
| **Social support** |  |  |  |  |  |  |  |  |  | 0.006 | -0.005 – 0.017 | 0.286 |
| **Quality of life** |  |  |  |  |  |  |  |  |  | -0.314 | -0.885 – 0.257 | 0.281 |
| **Depression** |  |  |  |  |  |  |  |  |  | -0.005 | -0.020 – 0.011 | 0.533 |
| **Observations** | 309 | | | 309 | | | 309 | | | 309 | | |
| **R^2^ / R^2^ adjusted** | 0.008 / 0.005 | | | 0.134 / 0.123 | | | 0.141 / 0.124 | | | 0.150 / 0.118 | | |
| ***High Education*** | | | | | | | | | | | | |
| **(Intercept)** | 5.236 | 4.839 – 5.633 | **<0.001** | 4.238 | 3.756 – 4.721 | **<0.001** | 4.135 | 3.650 – 4.620 | **<0.001** | 4.36 | 3.648 – 5.072 | **<0.001** |
| **Loneliness** | 0.005 | -0.003 – 0.012 | 0.244 | 0.005 | -0.003 – 0.012 | 0.218 | 0.004 | -0.003 – 0.011 | 0.306 | 0.003 | -0.005 – 0.010 | 0.476 |
| **Age** |  |  |  | 0.008 | 0.005 – 0.010 | **<0.001** | 0.008 | 0.006 – 0.011 | **<0.001** | 0.008 | 0.005 – 0.011 | **<0.001** |
| **Female** |  |  |  | 0.066 | -0.014 – 0.147 | 0.106 | 0.078 | -0.003 – 0.158 | 0.06 | 0.068 | -0.016 – 0.153 | 0.112 |
| **BMI** |  |  |  | 0.025 | 0.013 – 0.036 | **<0.001** | 0.025 | 0.013 – 0.036 | **<0.001** | 0.024 | 0.012 – 0.036 | **<0.001** |
| **Sitting time** |  |  |  |  |  |  | 0.017 | 0.005 – 0.029 | **0.007** | 0.018 | 0.005 – 0.030 | **0.006** |
| **Physical activity** |  |  |  |  |  |  | 0.003 | -0.001 – 0.007 | 0.100 | 0.003 | -0.000 – 0.007 | 0.082 |
| **Household income** |  |  |  |  |  |  |  |  |  | 0.000 | -0.000 – 0.000 | 0.148 |
| **Housemates** |  |  |  |  |  |  |  |  |  | 0.021 | -0.031 – 0.073 | 0.425 |
| **Social support** |  |  |  |  |  |  |  |  |  | -0.001 | -0.009 – 0.006 | 0.749 |
| **Quality of life** |  |  |  |  |  |  |  |  |  | -0.097 | -0.511 – 0.317 | 0.645 |
| **Depression** |  |  |  |  |  |  |  |  |  | 0.001 | -0.008 – 0.011 | 0.771 |
| **Observations** | 308 | | | 308 | | | 308 | | | 308 | | |
| **R^2^ / R^2^ adjusted** | 0.004 / 0.001 | | | 0.151 / 0.140 | | | 0.173 / 0.156 | | | 0.181 / 0.151 | | |

Table S3: Linear Regression Model of HbA1c (Subgroup for Physical activity)

|  | ***HbA1c (Model 1)*** | | | ***HbA1c (Model 2)*** | | | ***HbA1c (Model 3)*** | | | ***HbA1c (Model 4)*** | | |
| --- | --- | --- | --- | --- | --- | --- | --- | --- | --- | --- | --- | --- |
| ***Predictors*** | ***Est*** | ***(95% CI)*** | ***p*** | ***Est*** | ***(95% CI)*** | ***p*** | ***Est*** | ***(95% CI)*** | ***p*** | ***Est*** | ***(95% CI)*** | ***p*** |
| ***Physical Activity >= 6*** | | | | | | | | | | | | |
| **(Intercept)** | 5.590 | 5.200 – 5.980 | **<0.001** | 4.234 | 3.755 – 4.712 | **<0.001** | 4.341 | 3.829 – 4.853 | **<0.001** | 4.427 | 3.734 – 5.119 | **<0.001** |
| **Loneliness** | -0.001 | -0.009 – 0.007 | 0.781 | 0.001 | -0.006 – 0.008 | 0.878 | 0.000 | -0.007 – 0.007 | 0.941 | -0.000 | -0.007 – 0.007 | 0.990 |
| **Age** |  |  |  | 0.008 | 0.006 – 0.011 | **<0.001** | 0.008 | 0.005 – 0.010 | **<0.001** | 0.008 | 0.005 – 0.010 | **<0.001** |
| **Female** |  |  |  | 0.11 | 0.039 – 0.182 | **0.003** | 0.096 | 0.021 – 0.171 | **0.012** | 0.089 | 0.013 – 0.166 | **0.022** |
| **BMI** |  |  |  | 0.034 | 0.023 – 0.045 | **<0.001** | 0.034 | 0.023 – 0.045 | **<0.001** | 0.034 | 0.022 – 0.045 | **<0.001** |
| **Final education** |  |  |  |  |  |  | 0.001 | -0.047 – 0.050 | 0.957 | -0.005 | -0.056 – 0.046 | 0.845 |
| **Housemates** |  |  |  |  |  |  | -0.011 | -0.036 – 0.015 | 0.417 | -0.011 | -0.037 – 0.015 | 0.415 |
| **Household income** |  |  |  |  |  |  | 0.000 | -0.000 – 0.000 | 0.461 | 0.000 | -0.000 – 0.000 | 0.514 |
| **Social education** |  |  |  |  |  |  |  |  |  | 0.003 | -0.004 – 0.010 | 0.429 |
| **Quality of life** |  |  |  |  |  |  |  |  |  | -0.139 | -0.540 – 0.262 | 0.496 |
| **Depression** |  |  |  |  |  |  |  |  |  | 0.002 | -0.008 – 0.011 | 0.755 |
| **Observations** | 426 | | | 426 | | | 426 | | | 426 | | |
| **R^2^ / R^2^ adjusted** | 0.000 / -0.002 | | | 0.155 / 0.147 | | | 0.159 / 0.145 | | | 0.161 / 0.141 | | |
| ***Physical Activity < 6*** | | | | | | | | | | | | |
| **(Intercept)** | 5.777 | 5.042 – 6.511 | **<0.001** | 4.392 | 3.486 – 5.298 | **<0.001** | 4.647 | 3.660 – 5.635 | **<0.001** | 4.79 | 3.499 – 6.080 | **<0.001** |
| **Loneliness** | -0.005 | -0.019 – 0.010 | 0.513 | -0.004 | -0.017 – 0.010 | 0.594 | -0.002 | -0.016 – 0.012 | 0.77 | -0.001 | -0.015 – 0.013 | 0.89 |
| **Age** |  |  |  | 0.009 | 0.004 – 0.015 | **0.001** | 0.01 | 0.004 – 0.015 | **0.002** | 0.009 | 0.003 – 0.015 | **0.006** |
| **Female** |  |  |  | 0.049 | -0.100 – 0.198 | 0.515 | 0.006 | -0.150 – 0.163 | 0.936 | -0.004 | -0.170 – 0.162 | 0.965 |
| **BMI** |  |  |  | 0.035 | 0.014 – 0.056 | **0.001** | 0.028 | 0.006 – 0.049 | **0.011** | 0.028 | 0.006 – 0.049 | **0.013** |
| **Final education** |  |  |  |  |  |  | 0.042 | -0.064 – 0.148 | 0.437 | 0.043 | -0.068 – 0.153 | 0.445 |
| **Housemates** |  |  |  |  |  |  | -0.061 | -0.117 – -0.006 | **0.031** | -0.062 | -0.118 – -0.006 | **0.031** |
| **Household income** |  |  |  |  |  |  | 0.000 | -0.000 – 0.000 | 0.740 | 0.000 | -0.000 – 0.000 | 0.754 |
| **Social education** |  |  |  |  |  |  |  |  |  | 0.000 | -0.016 – 0.016 | 0.98 |
| **Quality of life** |  |  |  |  |  |  |  |  |  | -0.122 | -0.853 – 0.608 | 0.741 |
| **Depression** |  |  |  |  |  |  |  |  |  | -0.006 | -0.025 – 0.013 | 0.532 |
| **Observations** | 191 | | | 191 | | | 191 | | | 191 | | |
| **R^2^ / R^2^ adjusted** | 0.002 / -0.003 | | | 0.125 / 0.106 | | | 0.152 / 0.119 | | | 0.154 / 0.107 | | |

Appendix 1: Subgroup analysis result

In the group with income < 400 x 10^4^JPY (Table S1), after adjusting by co-variables in (model 2), (model 3), and (model 4), the association failed to be detected with all p-values > 0.05. In the group with income > 400 x 10^4^JPY, similarly to the cohort of income ≤ 400 x 10^4^JPY, after adjusting for co-variables, the association again failed to be detected with all p-values > 0.05. Table S2 shows the result of subgroup analysis when splitting the population into low and middle-education group and high-education group. After adjusting for co-variables in (model 2), (model 3), and (model 4), the association between loneliness and HbA1c has failed to detect, with all p-values > 0.05. Table S3 shows the linear regression analysis result on the group has physical activity level > 6 METs-h/day and ≤ 6 METs-h/day, respectively. After adjusting for co-variables in (model 2), (model 3), (model 4), the association between loneliness and HbA1c still has not been detected, all p-values > 0.05.

### *Sample size calculation*

According to the following equation^1^ and given the correlation between loneliness and HbA1c based on previous studies, at a confidence level of 95% and β=0.1, the sample size was estimated as shown in the following table.

$n={[(Z_{1}+ \frac{\alpha}{2}+Z_{1-\beta})/W]}^{2}+3$ (1)

In equation (1), W is calculated using the following equation:

$W=\frac{1}{2}\ln(\frac{1+r}{1-r})$ (2)

In equation (2), r is the estimated coefficient of correlation between loneliness and HbA1c in previous studies and assumptions. The correlation coefficient was reported as -0.09 in a study investigating the relationship between loneliness and blood glucose in diabetes^2^. Additionally, a study conducted in the US showed a positive correlation of 0.26^3^. Another study conducted in China reported the correlation coefficient between social isolation and glycosylated hemoglobin as 0.35^4^. Our study investigates the relationship between loneliness and HbA1c in the general population. With assumed correlation coefficient at 0.1, the required sample size would be 1046 participants.

Table S4: Sample size estimation based on assumption of correlation coefficient.

| Correlation coefficient | - 0.09 ^2^ | 0.1 (Assumed) | 0.26^3^ | 0.35^4^ |
| --- | --- | --- | --- | --- |
| Estimated sample size | 1293 | 1046 | 151 | 81 |

**References**

1. Negida A. Sample Size Calculation Guide - Part 7: How to Calculate the Sample Size Based on a Correlation. *Front Emerg Med*. 2020;4(2):e34-e34.

2. Kobos E, Szewczyk A, Świątkowska T, Kryczka T, Sienkiewicz Z. Relationship between loneliness and blood glucose control in diabetes. *BMC Public Health*. 2020;20(1):1140. doi:10.1186/s12889-020-09241-z

3. Huang YC, Cho E, Kuo HJ, García AA. The influences of depression and loneliness on A1C among middle-aged and older adults with diabetes. *Psychol Health Med*. Published online September 13, 2022:1-9. doi:10.1080/13548506.2022.2124287

4. Relationship between social isolation and glycaemic control of people previously diagnosed with diabetes: secondary analysis from the CHARLS | BMJ Open. Accessed May 29, 2024. https://bmjopen.bmj.com/content/14/3/e076106
